# Prism adaptation and spatial neglect: the need for dose-finding studies

**DOI:** 10.3389/fnhum.2015.00243

**Published:** 2015-04-30

**Authors:** Kelly M. Goedert, Jeffrey Y. Zhang, A. M. Barrett

**Affiliations:** ^1^Department of Psychology, Seton Hall UniversitySouth Orange, NJ, USA; ^2^Princeton PharmatechPrinceton, NJ, USA; ^3^Stroke Rehabilitation Research, Kessler FoundationWest Orange, NJ, USA; ^4^Department of Neurology and Neurosciences, Rutgers-New Jersey Medical SchoolNewark, NJ, USA; ^5^Department of Physical Medicine and Rehabilitation, Rutgers-New Jersey Medical SchoolNewark, NJ, USA

**Keywords:** prism adaptation, spatial neglect, dose-finding, inpatient rehabilitation

## Abstract

Spatial neglect is a devastating disorder in 50–70% of right-brain stroke survivors, who have problems attending to, or making movements towards, left-sided stimuli, and experience a high risk of chronic dependence. Prism adaptation is a promising treatment for neglect that involves brief, daily visuo-motor training sessions while wearing optical prisms. Its benefits extend to functional behaviors such as dressing, with effects lasting 6 months or longer. Because one to two sessions of prism adaptation induce adaptive changes in both spatial-motor behavior (Fortis et al., 2011) and brain function (Saj et al., 2013), it is possible stroke patients may benefit from treatment periods shorter than the standard, intensive protocol of ten sessions over two weeks—a protocol that is impractical for either US inpatient or outpatient rehabilitation. Demonstrating the effectiveness of a lower dose will maximize the availability of neglect treatment. We present preliminary data suggesting that four to six sessions of prism treatment may induce a large treatment effect, maintained three to four weeks post-treatment. We call for a systematic, randomized clinical trial to establish the minimal effective dose suitable for stroke intervention.

## Introduction

Spatial neglect is a devastating disorder, affecting 50–70% of individuals surviving right-hemisphere stroke (Paolucci et al., 2001; Buxbaum et al., 2004; Nijboer et al., 2013). The hallmark of the disorder is problems with attending to, or making movements towards, contralesional stimuli, not attributable to primary sensory or motor deficits (Heilman et al., 2011). Individuals presenting with neglect may have difficulty eating from the left side of their plate, dressing the left side of their body, or navigating their wheelchair towards the left (Heilman et al., 2011).

Relative to other stroke survivors, individuals with spatial neglect experience greater disability and poorer rehabilitation outcomes (Paolucci et al., 2001; Buxbaum et al., 2004; Gillen et al., 2005; Jehkonen et al., 2006). Their inpatient acute-care hospital stays are approximately 1.7 times longer than those without neglect (e.g., Kalra et al., 1997; Chen et al., 2015). They experience more in-hospital morbidity (e.g., more falls; Webster et al., 1995; Czernuszenko and Członkowska, 2009; Chen et al., 2015). They also experience poorer motor recovery, both during in-patient rehabilitation (e.g., Gillen et al., 2005; Chen et al., 2015) and in the months and years following stroke, even when neglect symptoms are remediated (e.g., Robertson et al., 1997; Nijboer et al., 2014; see Barrett and Muzaffar, 2014, for review). Because these individuals have a profoundly distorted sense of body-spatial relations (Riestra and Barrett, 2013), we might anticipate their problems with adaptive movements, transfers, balance and ambulation, as well as the association between neglect and decreased functional independence, decreased community mobility, and increased care-giver burden in chronic recovery (Buxbaum et al., 2004; Jehkonen et al., 2006; Oh-Park et al., 2014). Arguably the best setting for treatment of stroke is in-patient rehabilitation, where patients receive efficient and accessible care during the first days and weeks post-event, a critical period for neuroplastic brain change.

### Neural Mechanisms of Spatial Neglect

Neglect is associated with damage to any one of a number of structures in the frontal and parietal cortices, as well as with damage to the temporal-parietal junction, medial temporal, and subcortical sites, and white matter underlying fronto-parietal cortices (Doricchi and Tomaiuolo, 2003; Bartolomeo et al., 2007, 2012; Doricchi et al., 2008; Verdon et al., 2010; Thiebaut de Schotten et al., 2011). However, neglect is not a homogeneous disorder. It is likely that a subset of neglect symptoms, motor-intentional Aiming errors, are critically associated with the problems of motor recovery leading to functional dependence (Barrett and Muzaffar, 2014). These deficits can include directional hypokinesia (Barrett et al., 1999; Barrett and Burkholder, 2006), hemispatial hypokinesia (Hillis et al., 2006), and asymmetric perseveration (Khurshid et al., 2009). Growing evidence suggests that motor-intentional deficits stem from lesions of frontal cortex or its underlying white matter (e.g., Na et al., 1998; Ghacibeh et al., 2007; Verdon et al., 2010).

Importantly, unilateral lesions in these areas can produce *bilateral* hypoperfusion and hypoactivation of fronto-parietal networks, potentially exacerbating neglect (for a review see Vossel et al., 2014).

### Prism Adaptation as Promising Treatment for Improving Adaptive Action

Fortunately, a very promising treatment for neglect, prism adaptation, targets motor-intentional impairment and its neuro-anatomical pathways (Fortis et al., 2011; Saj et al., 2013), with long-lasting rehabilitative effects potentially lasting months to years (Fortis et al., 2010; Shiraishi et al., 2010; Mizuno et al., 2011). During prism adaptation treatment, individuals don prisms that displace their vision rightward and repeatedly perform a visually-guided, goal-directed action for approximately 20 min (e.g., Rossetti et al., 1998; see Redding and Wallace, 2006, for details). Individuals initially make errors in the direction of the visual displacement, but with repeated trials, become more accurate. Once the prisms are removed, adaptation is demonstrated by an aftereffect in which individuals make errors in the direction opposite the prism shift. For stroke survivors with left neglect, adapting to right-shifting prisms produces a leftward movement shift—they now make movements in the previously neglected left hemi-space. The benefits of prism adaptation extend to dressing, postural stability, walking, sit-to-stand transfers, and wheel-chair driving (Tilikete et al., 2001; Keane et al., 2006; Jacquin-Courtois et al., 2008; Shiraishi et al., 2010; Watanabe and Amimoto, 2010; see Jacquin-Courtois et al., 2013, for review).

Prism adaptation appears to exert its rehabilitative effects via action on the spatial-motor system (Striemer and Danckert, 2010; Fortis et al., 2011; Goedert et al., 2014). While “Where” perceptual-attentional unawareness is considered the hallmark of neglect, motor-intentional Aiming errors—also observed in spatial neglect—may be directly relevant to functional recovery (Heilman, 2004; Goedert et al., 2012; Barrett and Muzaffar, 2014). Fortis et al. (2011) administered two days of prism adaptation to five right-brain-damaged participants with spatial neglect. A computerized line bisection task allowing for separate quantification of Where and Aiming errors (Chen et al., 2011) demonstrated that all participants experienced improvement in spatial Aiming bias after prism adaptation, with no reliable improvement in perceptual-attentional Where errors. Furthermore, patients with spatial Aiming bias at baseline make greater functional gains after prism adaptation than those with only Where bias (Goedert et al., 2014).

In neglect, even a single session of prism adaptation leads to bilateral increases in task-specific activity in the middle frontal gyrus and superior parietal lobule (Saj et al., 2013). Thus, prism adaptation produces adaptive brain changes, potentially counteracting the bilateral hypoperfusion of frontal and parietal structures associated with unilateral lesions and neglect (Vossel et al., 2014). However, left and right medial temporal structures may mediate prism adaptation’s effects on neglect symptoms (Luauté et al., 2006; Chen et al., 2014). Nonetheless, with both left and right hemispheres participating in spatially-tuned movement, a bilateral increase in brain activity suggests prism adaptation may effectively modulate this system (see Barrett and Foundas, 2004 for a review; Hanna-Pladdy et al., 2001; Flores-Medina et al., 2014).

### What is the Appropriate Treatment Duration?

Although the low-risk, low-cost prism adaptation approach appears appropriate for broad use in acute care and rehabilitation, its feasibility is limited by lack of information about optimal dosing. No work has addressed the minimum effective dose for prism adaptation treatment. While prisms shifting the visual field at least 10° are likely necessary (Turton et al., 2010; Kerkhoff and Schenk, 2012; Mancuso et al., 2012; Fasotti and van Kessel, 2013), the minimal number of treatment sessions producing a *lasting* effect is not known.

From a rehabilitation standpoint, there is obvious necessity to demonstrate lasting effects of an intervention. Early studies of prism adaptation demonstrated immediate rehabilitative effects of a single session (see Barrett et al., 2012; Yang et al., 2013, for reviews). However, studies employing four or fewer once-daily prism treatments failed to find maintenance of that improvement a week (Farnè et al., 2002, after a single session) or month later (Nys et al., 2008, after four sessions). A minimum of two sessions a week may be necessary: Treating with prisms once a week for four weeks failed to produce a benefit (Rode et al., 2015). However, in a single case study, treating twice a week for nine weeks led to improvement that was sustained one year later (Humphreys et al., 2006). To date, studies demonstrating prism-related performance improvements lasting months to years have employed a minimum of ten prism treatment sessions (e.g., Fortis et al., 2010; Shiraishi et al., 2010). Indeed, the response to the lack of lasting improvement from four or fewer sessions has been a tacit move to a protocol of ten sessions, typically administered once daily over two weeks with weekends off (e.g., Frassinetti et al., 2002). By 2006, this tacit minimum standard was adopted by at least three major laboratories (e.g., Keane et al., 2006; Serino et al., 2006, 2007; Mizuno et al., 2011; Priftis et al., 2013).

A difficulty with this tacit standard is that U.S. inpatient rehabilitation facilities treat post-acute stroke patients for a length of stay of about 15 days (Dobson DaVanzo and Associates, 2014). During that time, all aspects of care must be managed, including patient and family education, training to use assistive devices, and treatments for all relevant medical conditions. If it takes two to three days to diagnose spatial neglect, patients may be discharged from inpatient rehabilitation before they have time to complete a ten-day/two-week prism protocol. An estimated 70 percent of stroke patients may not receive rehabilitation once discharged from inpatient care (Centers for Disease Control and Prevention (CDC), 2007). It is thus extremely important to make prism adaptation, and all intensive treatments, feasible for in-hospital administration.

As reviewed above, even one to two sessions of prism adaptation induce significant improvements in the cognitive and neural processes likely underlying prism adaptation’s beneficial effects (Fortis et al., 2011; Saj et al., 2013). Furthermore, a recent study of acute neglect suggests that the in-patient rehabilitation setting is very promising for prism treatment: Mizuno et al. (2011) treated mild and severe neglect patients undergoing inpatient rehabilitation within 12 weeks post-stroke, assessing patients’ improvement with the Behavioral Inattention Test-conventional (BIT-C), a neglect-specific assessment on which lower scores indicate poorer performance (Halligan et al., 1991). They found a large, positive effect of the prism treatment on the BIT-C among patients with mild neglect at a twelve week follow-up assessment. The difference between the prism-treated and control groups was substantial (Cohen’s *d* = 1.05), when compared with effect sizes of 0.3–0.6 often encountered in behavioral treatment studies. Although this same treatment benefit was not observed among the patients with severe neglect (because those in the severe neglect control group experienced an unusually large improvement), the large effect size in participants with mild neglect is promising.

Studies administering ten or more sessions of prism treatment produce lasting, and potentially large, effects (e.g., Mizuno et al., 2011). Studies administering four or fewer sessions of prism treatment do not detect lasting effects (e.g., Farnè et al., 2002; Nys et al., 2008). Might there be an intermediate number of prism treatment sessions that both produce a lasting effect and can be feasibly administered in the U.S. inpatient rehabilitation setting? Below we provide some preliminary data and estimate the effect size associated with an intermediate number of prism treatment sessions. These preliminary data suggest the importance of a true dose-finding study to evaluate shorter periods of prism treatment.

## Preliminary Data

In on-going studies in our laboratory, inpatient participants with neglect are randomized to a control condition (usual and standard rehabilitation) or to standard rehabilitation plus 10 days of once-daily prism treatment (11.3° right-shifting prisms). We assess their improvement with the BIT-C at study entry, immediately prior to the start of prism treatment, and weekly thereafter for five weeks. Thus, participants are assessed a total of seven times (T1 – T7), with prism adaptation treatment occurring between assessment time-points two (T2) and four (T4). Control participants experience the same weekly assessments without the prism treatment. As is common in longitudinal rehabilitation studies, there are patients who did not complete the full 10 sessions of once-daily prism treatment. We used this as an opportunity to perform exploratory data analyses investigating the potential effect size of a shorter treatment duration. Thus, we report only effect sizes, and not statistical significance.

This preliminary data, from two different studies of prism adaptation, contains five patients who were randomly assigned to a 0-prism control group and received only standard inpatient rehabilitation, as well as thirty participants who received prism treatment plus standard inpatient rehabilitation. Twenty-two of the 30 completed all ten prism treatment sessions, while eight completed between four and six prism-treatment sessions. These eight participants provided an opportunity to explore the effect size associated with a shorter treatment duration. There were no participants who received fewer than four sessions of treatment. The top three rows of Table 1 depict baseline characteristics of these three groups.

**Table 1:** **Characteristics of participants at baseline**.

Number of prism sessions	N	BIT-C	M/F	Age	Years education
0 prisms	5	102.8 (36.1)	3/2	63.2 (10.3)	13.6 (1.7)
4–6 prisms	8	80.4 (52.4)	6/2	64.9 (13.6)	12.1 (3.1)
10 prisms	22	88.18 (52.36)	16/6	61.9 (11.3)	14.9 (2.1)
Matched 10 prisms	8	79.3 (41.4)	6/2	61.9 (7.9)	16.0 (2.1)

*Note: Standard deviations in parentheses. Matched 10 prisms are 8 participants from the 10-prisms group matched via baseline BIT-C severity to individuals in the 4–6 prisms group*.

When assessing percent improvement over time, we used a conservative carry-forward method of imputing missing BIT-C data. Doing so affected 9 of the 245 assessment points or 3.6% of the data (3 time-points in 0-prisms group; 5 in 4–6 prisms; and 1 in 10 prisms). One potential problem for comparing the amount of improvement among these groups is that those receiving 4–6 prism sessions performed more poorly on the BIT-C at baseline than did the 0-prism control or 10-prism treatment group. Furthermore, we observed that percent improvement in BIT-C from pre- to post-prisms (i.e., T2 to T4) was negatively correlated with baseline BIT for prism-treated groups, but not for 0-prism controls (*r* = −0.92, *p* = 0.001, for 4–6 prisms; *r* = −0.66, *p* < 0.001, for 10 prisms; *r* = 0.19, *p* = 0.755, for 0 prisms). Thus, participants with more severe neglect experienced greater improvement with prism treatment. Because individuals in the 4–6 prisms group had more severe neglect (i.e., lower baseline BIT), we might spuriously observe disproportionate improvement in that group relative to the 10-prisms group.

Given this problem, we created a *matched* 10-prism group by selecting 8 of the 22 participants who were matched in baseline BIT-C severity to participants in the 4–6 prisms group. Baseline characteristics of this matched 10-prisms group appear in the last row of Table 1. Table 2 depicts the baseline BIT-C scores, as well as percent improvement from pre-prisms to post-assessment.

**Table 2:** **BIT-C scores and percent improvement over time**.

	Time 1	Time 2	Time 3 (Mid-Prisms)		Time 4 (Post-Prisms)		Time 7 (Last Follow-Up)	
	Baseline BIT-C	Pre-Prism BIT-C	% Improve	*d* vs. 0	% Improve	*d* vs. 0	% Improve	*d* vs. 0
0 prisms	102.8 (36.1)	102.0 (46.5)	0.70% (1.8)		3.3% (4.0)		3.0% (2.4)	
4–6 prisms	80.4 (52.4)	84.4 (54.7)	21.7% (28.0)	1.17	24.8% (38.5)	0.85	48.8% (62.3)	1.18
Matched 10 prisms	79.3 (41.4)	93.9 (43.0)	24.1% (29.1)	1.26	35.3% (82.3)	0.61	51.7% (115.0)	0.69

*Note: Standard deviations in parentheses. Mean BIT-C (Times 1 and 2) and % improvement in BIT-C (from Pre-Prisms/Time 2 to Time 3, 4, and 7). d = Cohen’s d for each treatment group vs. the 0-prism control group. Participants completed a total of 7 assessments, with one week occurring between each assessment. Participants completed daily prism adaptation treatment sessions starting at time 2 and concluding prior to time 4*.

As can been seen in Table 2, participants receiving only standard inpatient rehabilitation improved 3% from Time 2 to the last follow-up—a percent improvement comparable to that of the mild neglect control group of Mizuno et al. (2011). Over that same period, participants receiving 4–6 prism treatments or 10 prism treatments experienced greater, but similar, percent improvements of 48.8% and 51.7%, respectively. The Cohen’s *d* effect size for the difference between the respective treatment group and the 0-prism control was large for the 4–6 prisms group and medium for the matched 10-prisms group (due to larger variability in that group). Thus, these preliminary data suggest that even four to six prism treatment sessions may induce a large improvement in neglect that is maintained for a minimum of three to four weeks post-treatment.

## Discussion and Limitations

Our analyses suggest that four to six sessions of prisms may induce large treatment effects, lasting three to four weeks. However, caution must be exercised in interpreting these preliminary data. The groups do not result from a true randomization procedure. There is self-selection of individuals into our 4–6 prism group—i.e., some participants failed to complete the entire treatment protocol. Furthermore, the control and prism-treated groups in these preliminary data were not equivalent at baseline. Mizuno et al. (2011) found much greater improvement in their control group with severe as opposed to mild neglect. Our control group looks much like the Mizuno et al. (2011) mild control group, both in baseline severity and in improvement over time. However, Mizuno et al. (2011) observed group-level associations between percent improvement and baseline severity in their control groups, while we failed to observe a correlation between baseline severity and improvement over time in our control group. Thus, we do not think we would observe greater improvement in a control group matched for baseline severity to our two prism-treated groups.

Nonetheless, our goal here was not to provide definitive evidence that four to six sessions of prism treatment are sufficient. Rather, our goal was to establish the potential promise of a randomized clinical trial exploring the efficacy of fewer than 10 prism sessions for the treatment of neglect. Our results are indeed suggestive that fewer than 10 sessions may be effective for observing improvement on the BIT, a neglect-specific assessment. However, a true randomized clinical trial is strongly needed to definitively establish the minimum effective dose for producing sustained improvement on functional outcomes in addition to neglect-specific measures.

Furthermore, the data presented here highlight issues that may need to be addressed in a clinical trial, namely self-selection and variability in response to treatment. Among participants in our 4–6 prism group we observed not only a correlation between baseline severity and percent improvement, we also observed a positive correlation between the number of post-treatment assessment sessions completed and the percent improvement in BIT scores from T2 (pre-prisms) to T3 (mid-prism treatment; *r* = 0.67, *p* = 0.067, *n* = 8). This suggests that study participants demonstrating greater improvement may be more likely to adhere to and complete the study protocol.

The pattern of standard deviations observed in the percent improvement of prism-treated and control groups (Table 2) suggests variability in response to prism-treatment. A recent meta-analytic review also suggests large variability in response to prism treatment as assessed by the BIT (Yang et al., 2013). We previously observed that treatment with 10 prism sessions was more effective for improving functional performance in patients with spatial Aiming, motor-intentional symptoms, but not in patients with selective Where perceptual-attentional symptoms (Goedert et al., 2014). However, it is possible that individuals with selective Where perceptual-attentional deficits need higher doses—i.e., more prism adaptation sessions—to experience a treatment effect. We further observed that intact right medial temporal and sub-cortical structures could mediate a positive response to prism treatment (Chen et al., 2014). Thus, a clinical dosing trial would need to take into account each patient’s behavioral and neural profile, to address a possible interaction between neglect type and response to varying treatment duration.

## Conclusion

Streamlining a prism adaptation protocol and reducing treatment days by up to 50% could make an evidence-based regimen for prism training feasible in both inpatient and outpatient settings. Doing so would allow for broad implementation of this therapy, maximizing its ability to reduce the impact of stroke on severely affected survivors.

Given the promise of prism adaptation treatment, and its known, targeted effect on the critical neuroanatomical structures involved in the neglect disorder, it is time for systematic optimization of the dosage for prism adaptation in the treatment of neglect. In a larger sense, this issue also applies to a number of intensive, task-specific training methods that may be best administered during a critical period within weeks of stroke. For optimal transfer of research methods to the clinical setting, treatment duration needs to be prioritized as a major factor determining feasibility, cost, and applicability, so that systematic investigation of dosing contributes to protocols and clinical practice guidelines.

## Author Contributions

Authors AMB and KMG contributed to the design of the described studies. All authors participated in the analysis and interpretation of the data and in the drafting and revising of the manuscript’s intellectual content. All authors approved the final version and agree to be accountable for all aspects of the work.

## Conflict of Interest Statement

The authors declare that the research was conducted in the absence of any commercial or financial relationships that could be construed as a potential conflict of interest.
